# Biochar promotes compost humification by regulating bacterial and fungal communities

**DOI:** 10.3389/fmicb.2024.1470930

**Published:** 2024-09-18

**Authors:** Junying Zhang, Bowen Fan, Liqin Zhao, Changjiang Zhao, Fengjun Yang

**Affiliations:** ^1^College of Agronomy, Heilongjiang Bayi Agricultural University, Daqing, China; ^2^Key Laboratory of Low-Carbon Green Agriculture in Northeastern China, Ministry of Agriculture and Rural Affairs, Daqing, Heilongjiang, China; ^3^Engineering Research Center of Crop Straw Utilization, Heilongjiang Province, Daqing, Heilongjiang, China; ^4^College of Horticulture and Landscape Architecture, Heilongjiang Bayi Agricultural University, Daqing, China

**Keywords:** composting, biochar, humus, the cross-domain network, structural equation modeling

## Abstract

**Introduction:**

Humus can be formed during composting through biological pathways, nonetheless, the mechanisms through which bacterial and fungal communities govern the development of humus in compost with the addition of biochar remain uncertain.

**Methods:**

In this study, compost with cow dung and maize stover as feedstock was employed as a control group, and compost with 10% biochar added on top of the feedstock was adopted as a treatment group to investigate the effect of bacterial and fungal communities on humus formation during biochar composting.

**Results and Discussion:**

The results demonstrated that the humic acid content increased by 24.82 and 25.10% at the cooling and maturation stages, respectively, after adding biochar. Besides, the degree of polymerization content in the maturation stage was elevated by 90.98%, which accelerated the humification process of the compost. During the thermophilic and maturity stages, there was a respective increase of 51.34 and 31.40% in reducing sugar content, suggesting that the inclusion of biochar could furnish ample reducing sugar substrate for the Maillard reaction. The addition of biochar reduced the number of humus precursor-associated genera by 35, increased the number of genera involved in humus synthesis by two, and enhanced the stability of the cross-domain network between bacteria and fungi, which confirms that microorganisms contribute to the humification process by decreasing humus precursor consumption as well as increasing humus synthesis with the addition of biochar. Additionally, adding biochar could enhance the humification capacity of the compost pile by dominating the Maillard reaction with reducing sugars as the substrate and strengthening the function of humus synthesis-associated genera. This study enhances our comprehension of the regulatory pathways of biochar in the humification process during composting.

## 1 Introduction

Aerobic composting represents a synthetic humus technology, offering benefits such as cost-effectiveness, minimal pollution, and high efficiency. Besides, aerobic composting can convert macromolecular organic matter into humus through the action of microorganisms (Awasthi et al., 2019). There are three non-biological pathways for humus formation, namely the Maillard reaction pathway, the lignin-protein pathway, and the polyphenol pathway. Besides, the Maillard reaction is a polycondensation reaction that forms humus by utilizing reducing sugars and amino acids as humus precursors (Zhang and Tang, 2018). Meanwhile, the lignin-protein pathway is a polymerization reaction that forms humus by adopting quinones and amino acids as humus precursors (Pan et al., 2023). The polyphenol pathway involves a polycondensation process aimed at producing humus by utilizing polyphenols as the precursors for humus formation (Kastner et al., 1999). There are three biological pathways of humus formation, namely carbon fixation and humification, lignocellulose decomposition and humification, as well as denitrification and humification. Besides, the carbon fixation and humification pathway represents a mechanism through which microorganisms in compost enhance the production of humus precursors by capturing and sequestering carbon (Li et al., 2020). The lignocellulose decomposition and humification pathway serves as a way for microorganisms in compost to increase the content of humus precursors by decomposing lignocellulose (Jurado et al., 2015). Meanwhile, during composting, the denitrification and humification pathways facilitate the breakdown of aromatic compounds through denitrification. Consequently, this process increases the abundance of humus precursors, thereby fostering humus formation (Shi et al., 2020; Wu et al., 2020).

Both bacteria and fungi play an important role in the regulation of biological pathways in the compost humification process (Masigol et al., 2019). Moreover, Bacteria can promote organic matter degradation and increase the formation of humus precursors, facilitating the conversion of humus. Previous studies have demonstrated that the involvement of bacteria in humus precursors formation and the anabolism of aromatic substances contributes to the formation of redox functional groups in humus during composting (Zhao et al., 2020). In chicken manure compost, minerals can regulate the content of humus by influencing the composition and activity of bacterial communities (Yu et al., 2019). Extracellular enzymes secreted by fungi can mineralize and decompose macromolecular organic matter into humus precursors and also oxidize lignin to form humus (Cha et al., 2023; Sutradhar and Fatehi, 2023). Previous studies have illustrated that saprobic *basidiomycetes* can decompose complex organic matter into humus precursors by laccase and tyrosinase enzymes (Thorn et al., 1996). *Brown-rot* fungi can result in partial oxidation of lignin by peroxidase to form high molecular weight humus (Blanchette, 1991).

Feedstock properties, environmental conditions, as well as exogenous additives to aerobic composting affect the compost humification process (Wu et al., 2017a; Huang et al., 2019; Zhang et al., 2018). The introduction of manganese dioxide during composting stimulates humus formation through the conversion of amino acids and reducing sugars into humus (Zhang et al., 2020). Illite can increase the humus content of compost through biological and abiotic pathways, with the abiotic pathway of the Maillard reaction playing an essential role in increasing the humus content in the composting process (Pan et al., 2021). Furthermore, montmorillonite promotes humus formation in chicken manure compost by regulating the core bacterial community (Yu et al., 2019).

Biochar serves as a carbon-rich material produced by pyrolysis of biomass in a low-oxygen environment (Ahmad et al., 2014). Besides, the surface of biochar contains a porous structure and it contains a diverse array of functional groups, which have the characteristics of strong water-holding capacity, large specific surface area, and high CEC (Širić et al., 2023). Incorporating biochar into the composting process enhances both the quality and efficiency of compost by regulating pH levels, managing water content, improving carbon and nitrogen retention, and influencing the structure of microbial communities (Sánchez-García et al., 2015; Sanchez-Monedero et al., 2018). Biochar improves carbon and nitrogen retention in composting by adsorption of leachate and greenhouse gas emissions (Yin et al., 2021; Clough and Condron, 2010). Additionally, the loose and porous structure of biochar can provide a habitat for microorganisms, consequently influencing the community structure of microorganisms (Zhou et al., 2019). Meanwhile, the addition of biochar can regulate carbon metabolism by enhancing microbial degradation of organic carbon, stimulating the secretion of ligninase, which effectively degrades complex phenolic macromolecules, while concurrently suppressing the secretion of cellulase, which is capable of degrading polysaccharides (Luo and Gu, 2016). Biochar application accelerates microbial-mediated processes of nitrogen mineralization, nitrification, and denitrification. In doing so, it effectively modulates nitrogen metabolism (Liao et al., 2020). The intermediates formed by carbon and nitrogen metabolism, including amino acids, sugars, and nitrogenous compounds, are the precursor substances for the synthesis of humus (Motta and Santana, 2013). Biochar can regulate the humification process during composting (Wang et al., 2014). Nonetheless, the effects of the reactants of the Maillard reaction and the cross-domain communities of bacteria and fungi on the humification process under biochar conditions are still understudied.

In this study, mixed compost of cow dung and corn stover was employed as the control group. Biochar was added on top of the control group for the treatment group. Moreover, the primary goals of this research were as follows: (1) Determining the effect of biochar on humus precursors and humus fractions in the composting process; (2) Determining the effect of the cross-domain network of bacteria as well as fungi on the humification process of compost under biochar conditions; and (3) Modeling the role of biochar-driven humus precursors and humus synthesis-associated genera in the regulation of humification processes. The aforementioned analyses offer profound and innovative perspectives on how biochar facilitates humification during composting.

## 2 Materials and methods

### 2.1 Composting and sampling

All composting materials utilized in this experiment, along with their respective properties, were delineated in our prior investigation (Fan et al., 2024). This study was conducted from 23 July to 20 September 2022 (59d) at the experimental site of Heilongjiang Bayi Agricultural University (125°16′E, 46°59′N). The inclusion of maize stover was employed to adjust the C/N ratio of the compost feedstock to 25:1. The initial moisture content of the blend was adjusted to 65%. Moreover, the length × width × height of the pile was 2.5 m × 1.5 m × 1.0 m. To avoid compaction of the pile, manual turning of the pile was carried out at 6 d, 21 d, and 41 d. No biochar compost was the control group (CK), and the 10% biochar compost added on top of the control group was the treatment group (TB). Each treatment underwent replication three times.

Sampling was conducted according to the five-point sampling method during the initial (0 d), mesophilic (2 d), thermophilic (10 d), cooling (49 d), and maturation periods (59 d). Following thorough mixing, they were subdivided into two sub-samples: one stored at −80°C for analyzing bacterial and fungal diversity, and one air-dried and stored at −4°C for physicochemical index determination. The five stage samples were labeled CK0, CK2, CK10, CK49, CK59, TB0, TB2, TB10, TB49, TB59.

### 2.2 Analytical procedures

#### 2.2.1 Assessment of physicochemical indicators

Humus precursors were determined predominantly as amino acids (AA), reducing sugars (RS), total sugars (TS), and polysaccharides (PC) (Wu et al., 2017b). AA content was determined using an amino acid kit from *Solarbio* Science & Technology Corporation (Beijing, China). RS content was determined by 3, 5-dinitrosalicylic acid (DNS) method by mixing the sample with DNS reagent in a boiling water bath and subsequently measuring absorbance at 540 nm. Furthermore, TS content was determined by phenol-sulfuric acid method, and the specific method was to mix the sample with 5% phenol solution, add concentrated sulfuric acid for 20 min, and measure the absorbance at 490 nm wavelength (Fadel et al., 2018). The PC content was determined by calculating the difference between the TS content and the RS content (Si et al., 2019).

Humus can be divided into two components based on molecular weight, humic acid (HA) and fulvic acid (FA). Using the method of Zhou et al. (2014), the air-dried samples were mixed with the extraction solution (mixed with 0.1 M sodium pyrophosphate Na_4_P_2_O_7_ and NaOH) at a solid–liquid ratio (w/v) of 1:20, and the filtrate was collected by shaking at 200 rpm for 24 h at 25°C and centrifuging at 12,000 rpm for 15 min. The obtained filtrate was mixed with 6 M HCL to give the humus solution a pH of 1 and centrifuged at 12,000 rpm for 15 min. FA was obtained upon filtration through a 0.45 μm filter, the precipitate was washed several times with 0.05 M HCI and subsequently HA was obtained by dissolving it with 0.05 M NaHCO_3_. The degree of polymerization (DP) and humification index (HI) were calculated based on the following equation (Wu et al., 2017b):


$$DP=\frac{CHA}{CFA}$$

$$HI=\frac{CHA}{TOC}\times 100$$

#### 2.2.2 DNA extraction and pyrosequencing

Extraction of DNA from compost samples using Omega Bio-tek Soil DNA Extraction Kit (Omega Bio-tek, Inc., USA). The V3-V4 region of the bacteria 16S rRNA gene was amplified by employing the following primer pair: 338F, ACTCCTACGGGAGGCAGCAG, and 806R, GGACTACHVGGGTWTCTAAT. The ITS1 region was amplified using the specific fungal primers ITS1-F, CTTGGTCATTTAGAGGAAGTAA, and ITS2, GCTGCGTTCTT CATCGATGC. Additionally, the purified amplicons were then sequenced using an Illumina MiSeq PE300 (Allwegene Tech. Beijing, China).

#### 2.2.3 Sequence process

The raw data underwent quality control and were merged using Pear (v0.9.6). Chimeric sequences from the trimmed and filtered data were identified and removed with VSEARCH (v2.7.1). OTU clustering was also conducted using VSEARCH (v2.7.1). The representative sequences of bacterial OTUs were aligned against the Silva138 database using the BLAST algorithm to acquire bacterial species annotation data. Similarly, the representative sequences of fungal OTUs were aligned with the Unite8.2 database using the BLAST algorithm to obtain fungal species annotation information. The original sequence was stored in the NCBI Sequence Read Archive (SRA), and the Bioproject number is PRJNA1105238.

#### 2.2.4 Statistical analysis

Data were presented as means and standard deviations of three replicate samples and were compared by ANOVA and Duncan’s multiple comparison test in SPSS 25.0 (IBM, USA) (*p* < 0.05). Besides, Graphs were constructed with GraphPad Prism (8.0.1.244). The “plot(g)” software package was used to process the cross-domain network data and the Gephi software was employed to assess and visualize the network topology. Network analysis utilizing Pearson’s correlation coefficient identified key bacterial and fungal genera correlated with humus precursors and humus indices. Structural equation modeling (SEM) was conducted using AMOS 23.0 to evaluate hypothetical pathways elucidating the impact of biochar on humification indices. Overall SEM goodness-of-fit was based on a Chi-square test (*χ*^2^ < 10) and non-significant Chi-square tests (*p* > 0.05).

## 3 Results and discussion

### 3.1 Effect of biochar addition on humus fractions of composts

**Figure 1:**
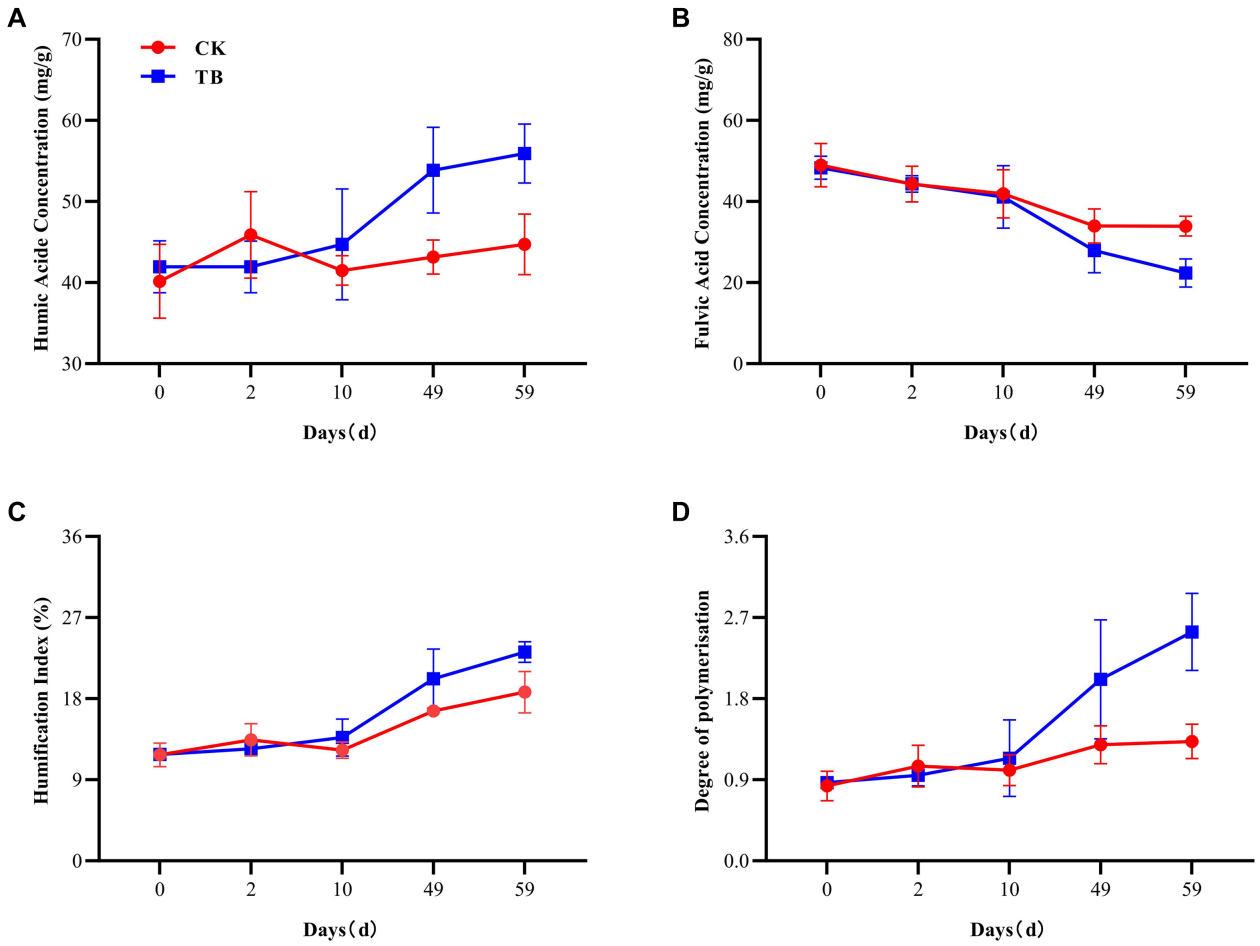
The humification process of composting. **(A)** Humic acids concentration. **(B)** Fulvic acids concentration. **(C)** Humification index. **(D)** Degree of polymerization. CK, and TB denote the treatments without biochar, with added 10% biochar, respectively. Values are illustrated as means ± standard deviations (*n* = 3).

HA and FA are both essential components of humus (Collado et al., 2018). HA has a large molecular weight and is highly stable; FA possesses a lower molecular weight and is susceptible to oxidation (Tan and Giddens, 1972). Besides, the HA content of TB treatment was substantially higher than that of control at cooling and maturation stages, elevated by 24.82 and 25.07%, respectively (Figure 1A), which may be since the biochar itself has functional groups that bind to FA and promote the formation of HA (Cybulak et al., 2021). FA content demonstrated a decreasing trend in both TB and CK, implying that FA polymerization was promoted with composting (Figure 1B). The content of FA in the TB at maturation was significantly lower than that of CK, by 51.68%. The reason may be that the addition of biochar promotes the polymerization of FA, resulting in the formation of more HA.

HI and DP indicate the stability and maturity of humification, respectively (Wu et al., 2017b). In both TB and CK treatments, the levels of HI and DP exhibited an upward trajectory (Figures 1C,D). At the maturation stage, HI and DP experienced a notable increase in the TB treatment, by 23.71 and 90.98%, respectively, compared to the CK treatment. This observation suggests that the compost’s humus possesses a more stable structure and maturity following the addition of biochar, potentially attributable to the conversion of FA to HA.

### 3.2 Effect of biochar addition on humus precursor of composts

**Figure 2:**
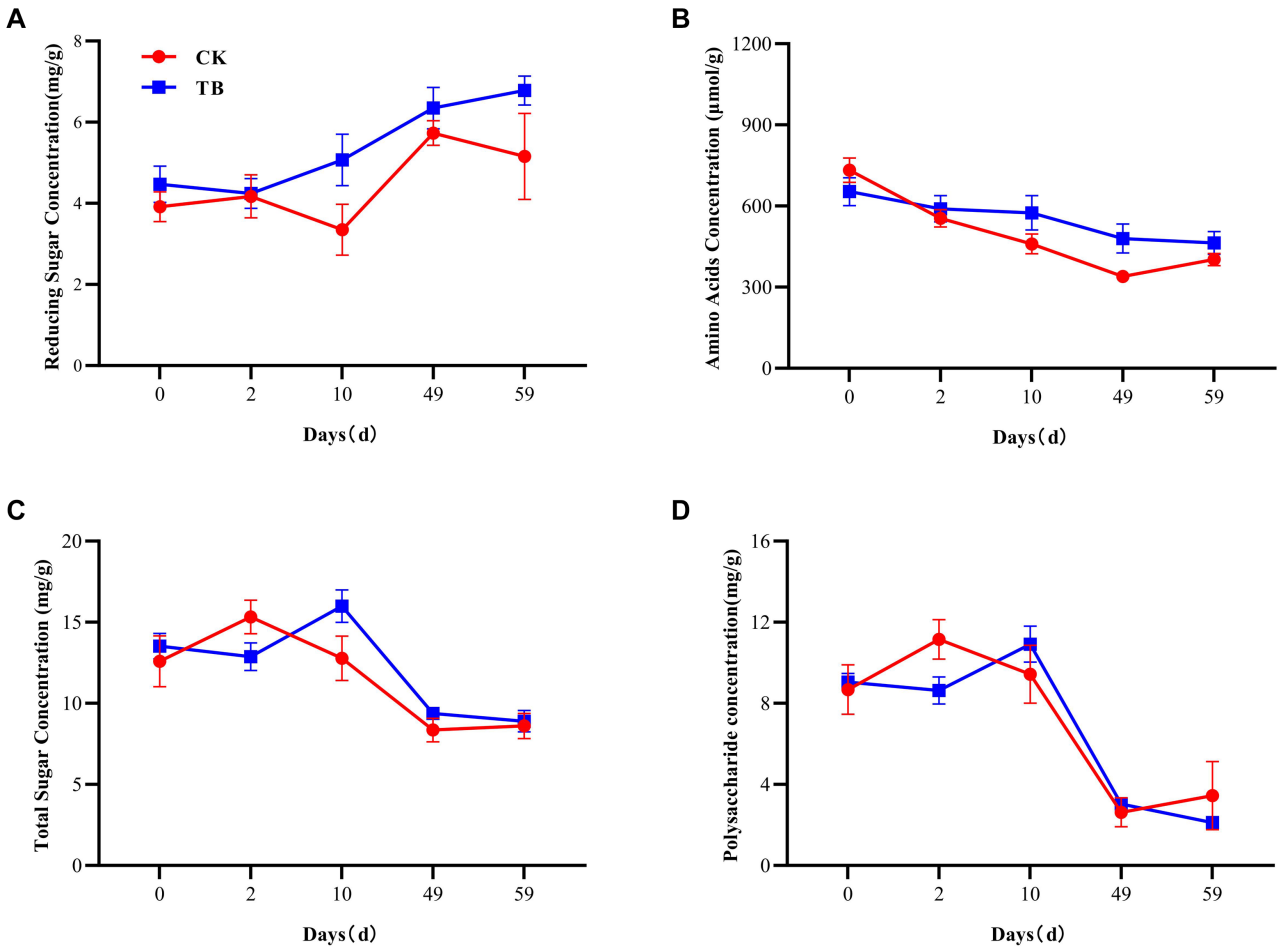
Changes in various precursors concentration during composting. **(A)** Reducing sugar concentration. **(B)** Amino acids concentration. **(C)** Total sugar concentration. **(D)** Polysaccharide concentration. CK and TB refers to the treatments lacking biochar and those incorporating 10% maize straw biochar, correspondingly. Values are indicated as means ± standard deviations (*n* = 3).

RS and AA are precursors for the production of humus by the Maillard reaction. Moreover, the TS and PC contents affect the RS content (Chen et al., 2008; Jokic et al., 2001). There was an overall increasing trend in the RS content of TB (Figure 2A). At thermophilic and maturation stages, the RS content of TB was considerably higher than that of CK, by 51.34 and 31.40%, respectively. The addition of biochar promotes increased levels of RS, which may be attributable to the enhanced mineralization of organic matter resulting from the addition of biochar (Lehmann et al., 2021). It provided sufficient substrate for the synthesis of HA. AA content indicated a decreasing trend in both CK and TB. AA content of TB was considerably higher than that of CK during thermophilic and cooling periods, which were elevated by 25.08 and 41.44%, respectively (Figure 2B). Moreover, the addition of biochar indirectly provided sufficient precursors for the Maillard reaction. This process was facilitated by both the thermophilic and cooling phases, which play crucial roles in the formation of humus (Wu et al., 2020). Both TS and PC revealed a tendency to increase and then decrease during the composting process (Figures 2C,D), which indicates that TS and PC were consumed substantially during the composting process. The TS and PC contents of the TB were considerably higher than that of CK during the thermophilic period, by 19.11 and 29.32%, respectively, which indicates that biochar can increase the TS and PC content in the thermophilic period, and enhance sufficient security for RS to participate in the Maillard reaction. This could be attributed to the enhancement of microbial activity resulting from the addition of biochar during the composting process. The heightened microbial activity accelerates the decomposition and mineralization of organic matter (Wu et al., 2017b).

### 3.3 Effects of biochar addition on bacterial and fungal communities

**Figure 3:**
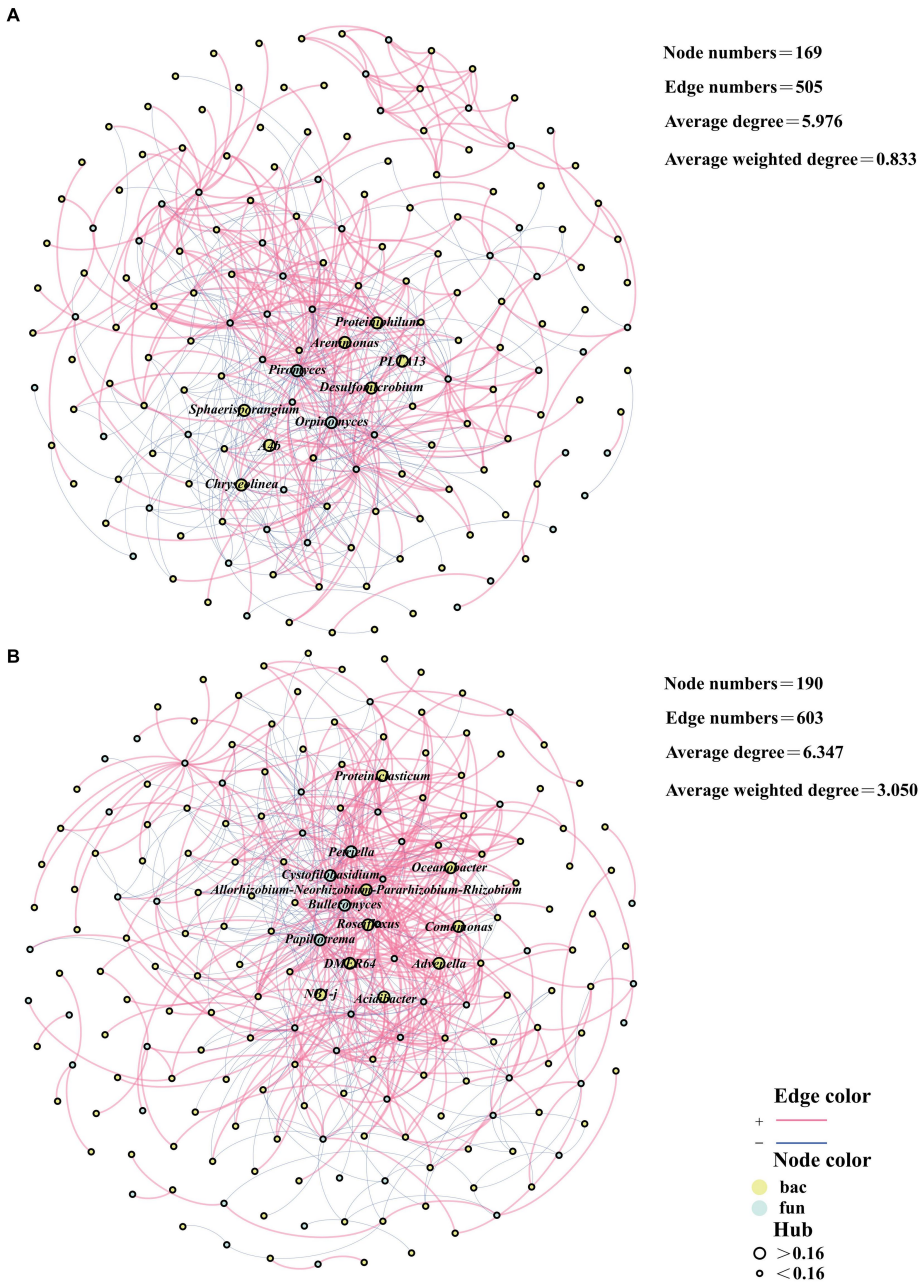
Network analysis based on the bacterial and fungal cross-domain networks of CK composting **(A)** and TB composting **(B)**. CK and TB denote the treatments without biochar, with added 10% maize straw biochar, respectively. Besides, red lines represented the significantly positive correlations, while blue lines represented the significantly negative correlations. Yellow nodes represented bacteria, while green nodes indicated fungi. Nodes with hub values greater than 0.16 are denoted by large nodes, while small nodes denote Nodes with hub values less than 0.16.

The cross-domain network analyses of bacteria and fungi can depict the interplay between bacterial and fungal communities during the composting process (Gao et al., 2024). In CK, the bacterial and fungal cross-domain network had a cumulative total of 169 edges and 505 points. Besides, the average degree of the network was 5.976 and the average weighted degree was 0.833 (Figure 3A). In the TB group, the cross-domain network of bacteria and fungi had a combined total of 190 edges and 603 nodes. The average degree of the network was 6.347, while the average weighted degree was 3.050 (Figure 3B). The number of edges and points in the bacterial and fungal cross-domain networks of the TB was higher than that of CK, implying that the complexity of the network of the TB was higher than that of CK. The average degree and average weighted degree of the bacterial and fungal cross-domain networks of TB were higher than that of CK, indicating that the clustering coefficient of the network of TB was higher than that of CK. This phenomenon could be attributed to the strengthening of the interaction relationship between bacteria and fungi resulting from the addition of biochar.

Hub microorganisms are key nodes in the network and play a pivotal role in the network. Hub microorganisms typically support the stability of the network (Li et al., 2023). In the cross-domain network, there were nine hub microorganisms in CK, including seven bacteria, *Proteiniphilum*, *Arenimonas*, *PLTA13*, *Desulfomicrobium*, *Sphaerisporangium*, *A4b*, and *Chryseolinea*, and two of which were fungi, *Piromyces*, *Orpinomyces*. There were 13 hub microorganisms in the TB, including nine bacteria, *Proteiniclasticum*, *Oceanobacter*, *Allorhizobium-Neorhizobium-Pararhizobium-Rhizobium*, *Roseiflexus*, *Comamonas*, *DMER64*, *Advenella*, *NB1-j*, *Acidibacter*, and four fungi, *Petriella*, *Cystofilobasidium*, *Bulleromyces*, *Papiliotrema*. The addition of biochar elevated the count of hub microorganisms in the cross-domain network of bacteria and fungi, in which the number of both bacteria and fungal hub microorganisms increased. This suggests that both bacteria and fungi assume key roles as the stability of the network following the addition of biochar.

### 3.4 Relationship between humus and bacteria with fungi in composting

**Figure 4:**
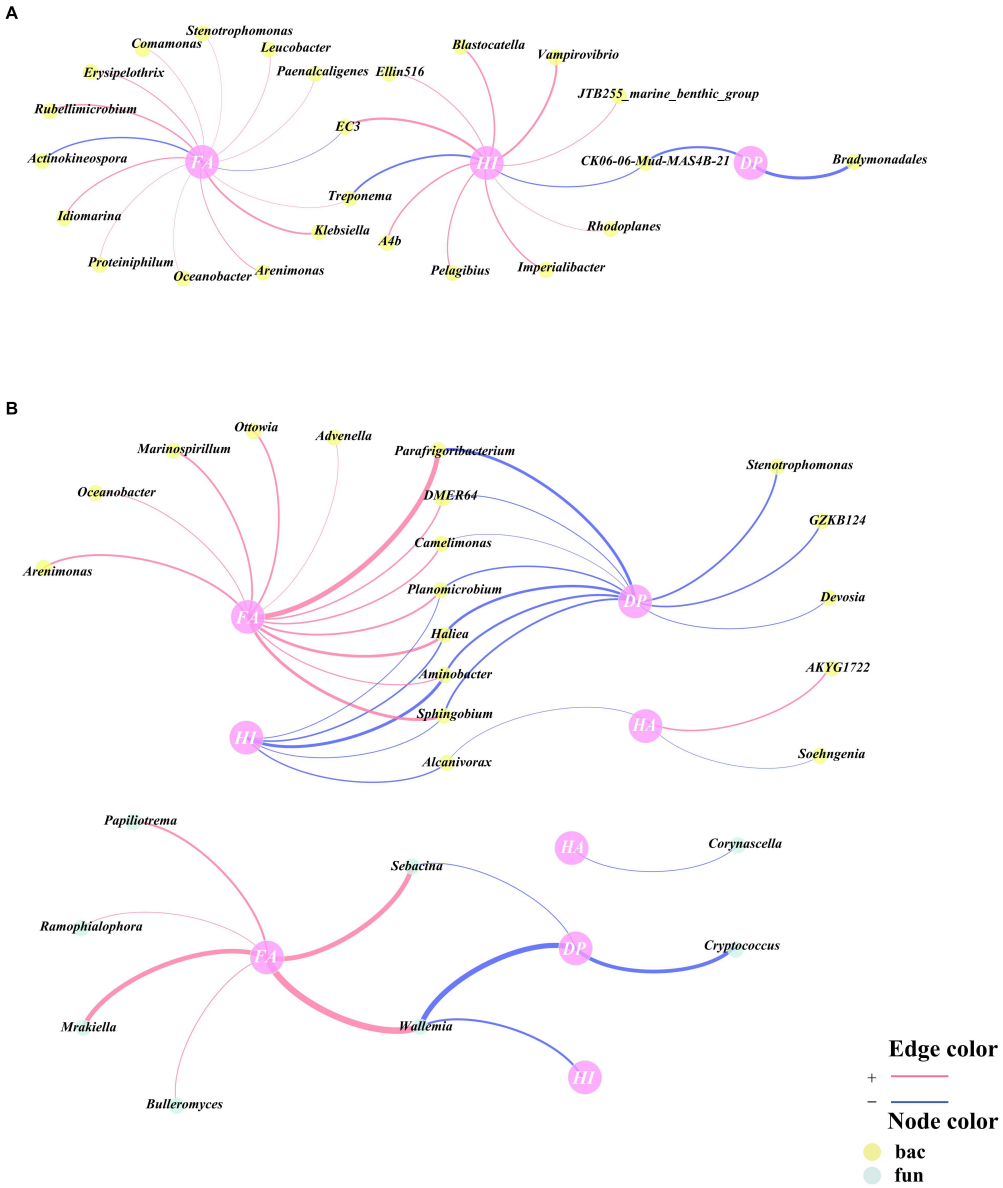
Network analysis of key bacterial and fungal related to humic acids (HA), fulvic acids (FA), humification index (HI), and degree of polymerization (DP) according to Pearson correlation (*p* < 0.05) for CK **(A)** and TB **(B)** treatment in composting. CK and TB imply the treatments without biochar, with added 10% biochar, respectively. The red lines indicated statistically significant positive correlations, whereas the blue lines indicated statistically significant negative correlations. Yellow nodes denoted bacteria, while green nodes denoted fungi.

Pearson correlation analyses of HA, FA, HI, and DP with genus-level bacteria and fungi were performed correlation networks were constructed, and the genera within the correlation networks were classified as being associated with humus synthesis. A total of 24 genera were associated with humus synthesis in CK, all of which were bacterial genera (Figure 4A). Moreover, a total of 26 genera were in association with humus synthesis in TB, of which 18 were bacterial genera and 8 were fungal genera (Figure 4B), which indicates that more fungi are involved in humus synthesis following the addition of biochar. This could be attributed to the heightened significance of fungi in regulating the biological pathways involved in the humification process following the addition of biochar. The functional groups and electron transfer characteristics of biochar itself promoted the participation of fungi in humus synthesis, which is consistent with the findings of Ma et al. (2024).

Among humus synthesis-associated genera of CK, three bacteria (*Arenimonas*, *Proteiniphilum*, *A4b*) were hub microorganisms in the cross-domain network (Figures 3A, 4A). Among humus synthesis-associated genera of TB, three bacteria (*Oceanobacter*, *Advenella*, *DMER64*) and two fungi (*Papiliotrema*, *Bulleromyces*) were hub microorganisms in the cross-domain network (Figures 3B, 4B). This phenomenon also implies, from another angle, that genera associated with humus synthesis assume a more prominent role in the cross-domain network of bacteria and fungi following the addition of biochar.

### 3.5 Relationship between humus precursors and bacteria with fungi in composting

**Figure 5:**
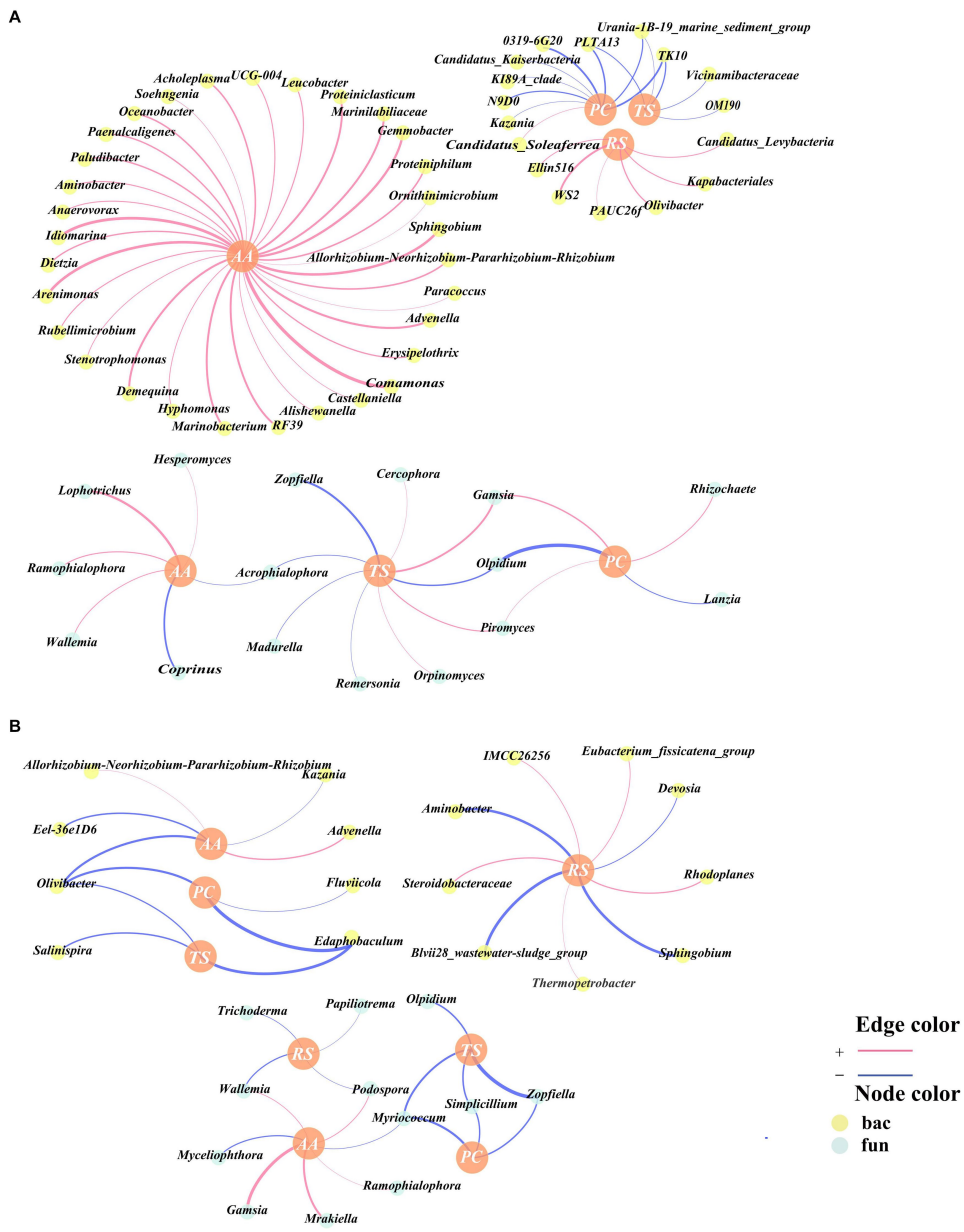
Network analysis of key bacterial and fungal related to reducing sugar (RS), amino acids (AA), total sugar (TS), and polysaccharide (PC) according to Pearson correlation (*p* < 0.05) for CK **(A)** and TB **(B)** treatment in composting. CK and TB denote the treatments without biochar, with added 10% biochar, respectively. Red lines demonstrated significantly positive correlations, while blue lines indicated significantly negative correlations. Yellow nodes represented bacteria, while green nodes represented fungi.

Pearson correlation analyses were conducted on RS, PC, TS, and AA with bacteria and fungi at the genus level, and correlation networks were established. The genera present in these correlation networks were identified as being associated with humus precursors. A total of 64 genera were associated with humus precursors in CK, including 48 bacteria and 16 fungi (Figure 5A). In TB, there were 29 genera associated with humus precursors, namely, 17 bacteria and 12 fungi (Figure 5B). In the TB group, there were fewer bacteria and fungi associated with humus precursors compared to the CK group. On the one hand, bacteria and fungi do not directly participate in the Maillard reaction to synthesize humus, and on the other hand, bacteria and fungi can consume humus precursors to conduct their physiological activities (Wu et al., 2017b). It is hypothesized that bacteria and fungi in CK consume a large amount of humus precursors, and consequently weakening the Maillard reaction. The addition of biochar resulted in a decrease in the consumption of humus precursors by bacteria and fungi, thereby facilitating the Maillard reaction. There were 37 and 12 genera associated with AA in CK and TB, respectively, and there were 6 and 13 genera associated with RS in CK and TB, respectively. More genera were associated with AA in CK and more genera were associated with RS in TB, which indicates that biochar addition changed the utilization relationship between bacteria and fungi and humus precursors, where bacteria and fungi transitioned from metabolizing AA to metabolizing RS.

### 3.6 Effect of biochar addition on bacterial and fungal community stability

**Figure 6:**
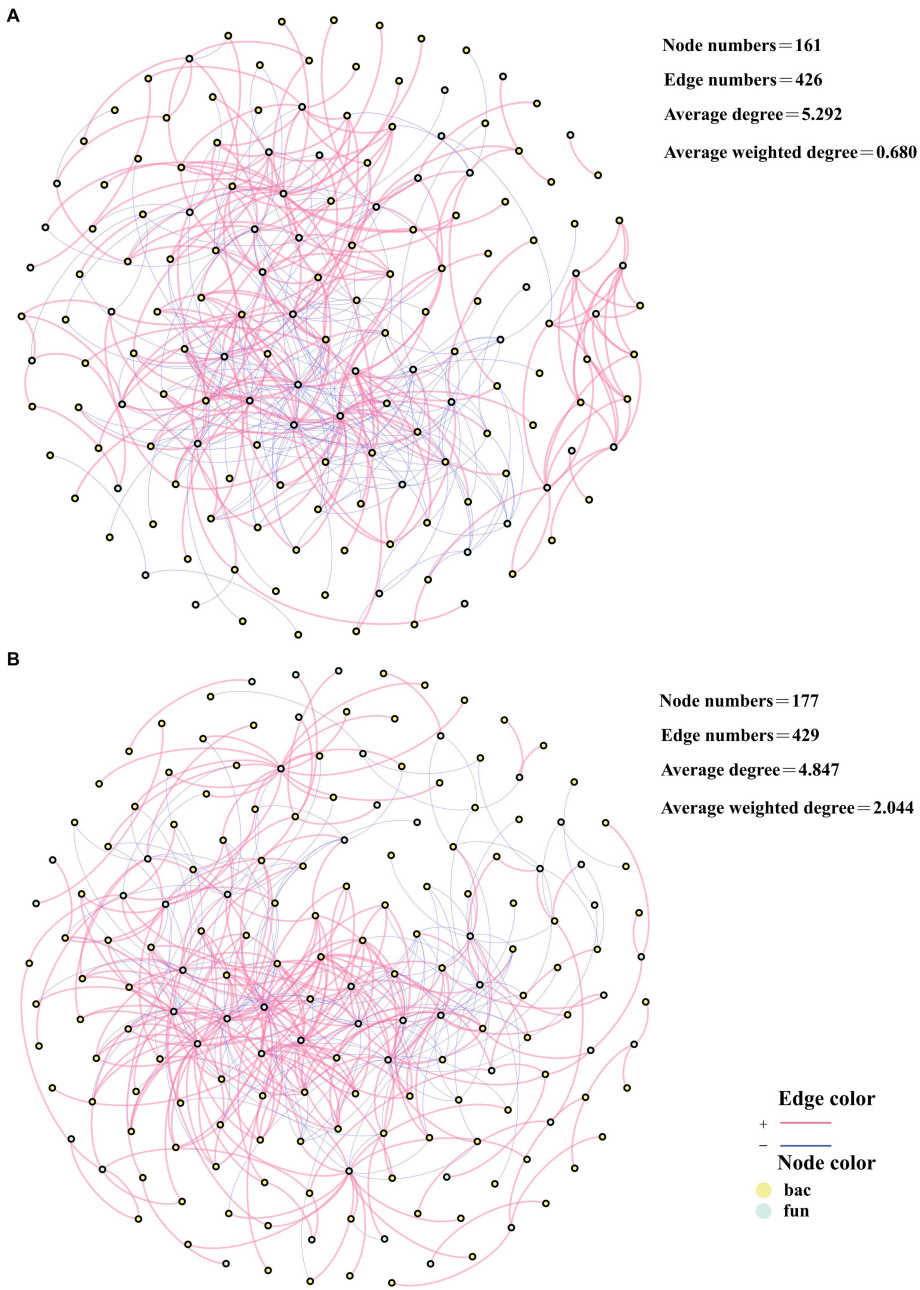
Network analysis of destabilized bacterial and fungal cross-domain networks based on destabilization of CK compost **(A)** and TB compost **(B)**. CK and TB denote the treatments without biochar, with added 10% biochar, respectively. Red lines denoted significant positive correlations, while blue lines indicated significant negative correlations. Yellow nodes were indicative of bacteria, whereas green nodes represented fungi.

To investigate the effect of hub microorganisms on network stability, the network was reconstructed by removing all hub microorganisms from the cross-domain network, and the alteration in network stability was evaluated by examining the key topological properties of the reconstructed network. In CK, the destabilized bacterial and fungal cross-domain network had a total of 161 edges and 426 points. Besides, the average degree of the network was 5.292 and the average weighted degree was 0.680 (Figure 6A). In TB, the destabilized bacterial and fungal cross-domain network had a total of 177 edges and 429 points, and the average degree of the network was 4.847 and the average weighted degree was 2.044 (Figure 6B). In comparison with the normal bacterial and fungal cross-domain network, the destabilized bacterial and fungal cross-domain network had 8 fewer edges and 79 fewer points in CK and 13 fewer edges and 174 fewer points in the TB. This suggests that the removal of hub microorganisms decreases network stability in TB compared to CK. Among the deleted hub microorganisms in CK, three were associated with humus synthesis (Figures 3A, 6A). In contrast, in TB, five deleted hub microorganisms were associated with humus synthesis (Figures 3B, 6B). It was further verified that humus synthesis-associated genera had an essential position in the bacterial and fungal communities after the addition of biochar.

### 3.7 Relationship between humus fractions and environmental factors after biochar addition

**Figure 7:**
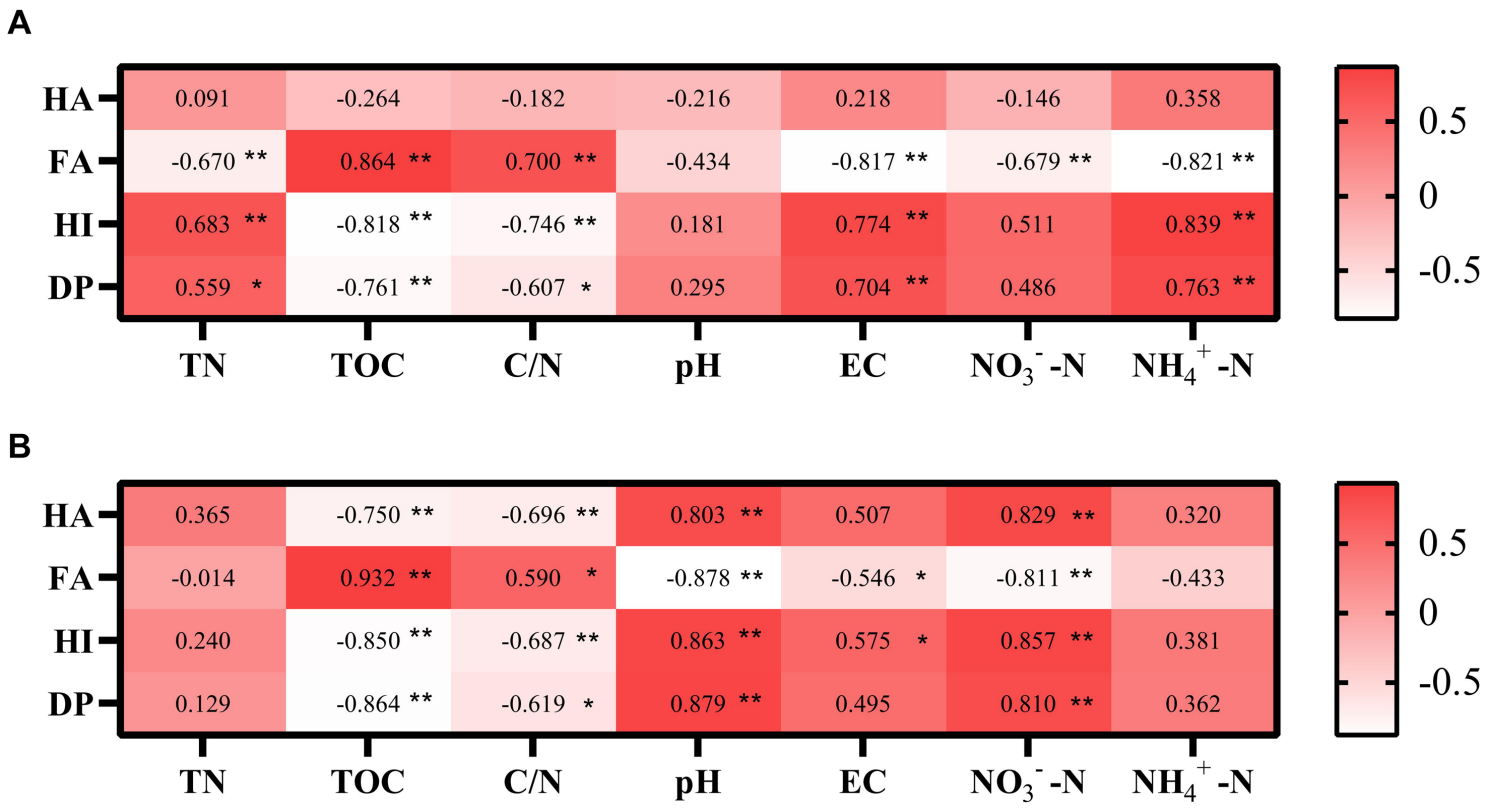
Pearson correlation analyses of associations between the humification and the chemical property of CK compost **(A)** and TB compost **(B)**. CK and TB indicate the treatments without biochar, with added 10% biochar, respectively. *0.01 < p < 0.05, **0.001 < p < 0.01.

Spearman correlation analysis was used to explore the relationship between humus fractions and environmental factors. Besides, in CK, HA, FA, HI, and DP were not substantially correlated with pH (Figure 7A). In the TB, HA, FA, HI, and DP were all considerably correlated with pH (Figure 7B). This indicates that biochar addition will alter the humification process by regulating the pH, which may be due to the alkaline nature of biochar itself. FA correlated significantly with both nitrate nitrogen in CK. HA, FA, HI, DP, and all significantly correlated with nitrate nitrogen in TB, which suggests that nitrate nitrogen with the addition of biochar can regulate humus stability and maturation. FA, HI, and DP correlated markedly with C/N in CK. HA, FA, HI, and DP were all significantly correlated with C/N in TB, which illustrates that C/N with or without biochar addition affects the humification process. The C/N under biochar addition promotes the formation of large molecular weight humus.

### 3.8 Identifying pathways for biochar-driven humification processes based on SEM

**Figure 8:**
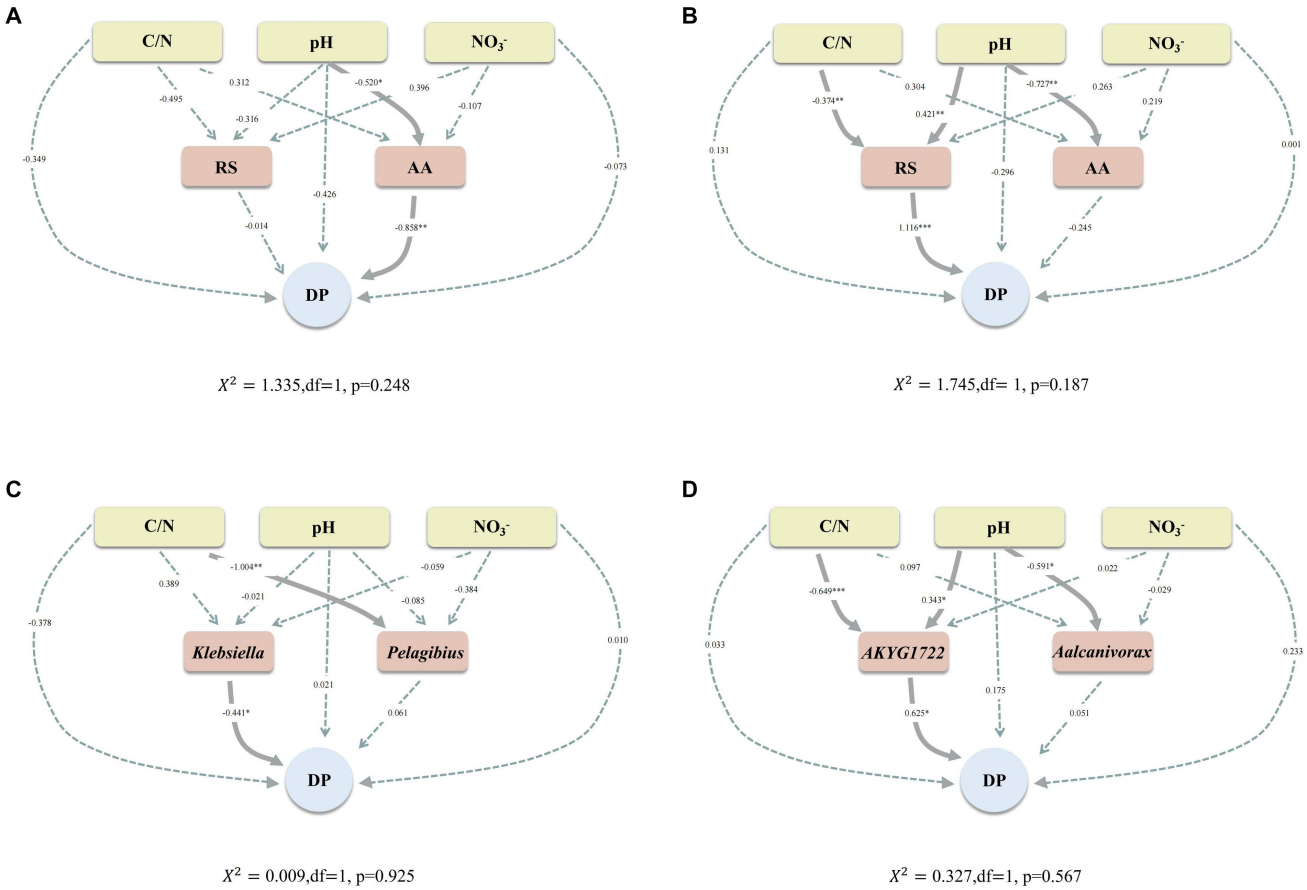
**(A,B)** Structural equation models developed to explain CK composting **(A)** and TB composting **(B)**, highlighting hypothetical causal associations between particular chemical properties, substrate for the Maillard reaction, and DP. **(C,D)** Structural equation models were developed to explain CK composting **(C)** and TB composting **(D)**, highlighting hypothetical causal associations between particular chemical properties, humus synthesis-associated genera, and DP. CK and TB represent the treatments devoid of biochar and those enriched with 10% biochar, respectively. Solid and dashed arrows signify significant and non-significant causal relationships, respectively. Numbers adjacent to arrow paths indicate path coefficients. *0.01 < *p* < 0.05, **0.001 < *p* < 0.01, ***p < 0.001.

For the purpose of verifying that the Maillard reaction (abiotic pathway) and humus synthesis-associated genera (biotic pathway) can promote the humification process. Besides, the relationship between C/N, pH, NO3^−^, RS, AA, *Klebsiella*, *Pelagibius*, *AKYG1722*, *Aalcanivorax*, and DP was assessed by utilizing SEM. In both CK and TB, pH exerted a pronounced and direct influence on the content of AA (Figures 8A,B). This suggests that pH exerts a consistent impact on AA content during composting, irrespective of biochar addition. Moreover, in CK, pH was found to significantly influence the DP via its effect on AA content. In TB, C/N and pH can significantly affect DP through RS content, which implies two changes in the pathways regulating humification maturity with the addition of biochar. In the first place, switching from a pH-regulated humification maturity pathway to a C/N and pH-regulated humification maturity pathway; secondly, transitioning from a pathway dominated by AA in the Maillard reaction to a pathway dominated by RS in the Maillard reaction. These two switched regulatory pathways may be the predominant reason why biochar increases humification maturity in compost. The C/N ratio can influence the maturity of humification with the addition of biochar. This effect may arise from biochar’s regulation of the C/N ratio through microbial denitrification processes (Li et al., 2024).

In CK, *Klebsiella* could directly and considerably affect DP (Figure 8C), which indicates that humus synthesis-associated genera can influence humification maturity. In TB, C/N and pH could significantly affect DP by *AKYG1722* (Figure 8D). This implies that after the addition of biochar, the C/N ratio and pH levels promote the humification process by regulating genera associated with humus synthesis. Meanwhile, this may be the main biological pathway by which biochar affects the humification process. This phenomenon may be attributed to the binding of bacteria to the surface of the biochar, which acts as an electron shuttle and facilitates the humification process (Dong et al., 2023).

## 4 Conclusion

The significance of this study extends to the pathways by which biochar regulates the humification process—evaluating the role of biochar in regulating the Maillard reaction and microbial communities. The study determined biochar was discovered to contribute to the humification process during composting. Moreover, in the abiotic pathway of biochar-regulated humus, elevated levels of RS in the Maillard reaction contribute to the humification process in the abiotic pathway. In the biological pathway of biochar-regulated humus, the augmentation of centrality among genera associated with humus synthesis in the cross-domain network of bacteria and fungi contributes to the humification process within this biological pathway. Based on these findings, future research should explore the effects of distinct biochar materials and environmental conditions on humification processes.

## Data Availability

The datasets presented in this study can be found in online repositories. The names of the repository/repositories and accession number(s) can be found in the article/supplementary material.
